# Prediction of Lung Nodule Progression with an Uncertainty-Aware Hierarchical Probabilistic Network

**DOI:** 10.3390/diagnostics12112639

**Published:** 2022-10-31

**Authors:** Xavier Rafael-Palou, Anton Aubanell, Mario Ceresa, Vicent Ribas, Gemma Piella, Miguel A. González Ballester

**Affiliations:** 1 BCN MedTech, Department of Information and Communication Technologies, Universitat Pompeu Fabra, 08108 Barcelona, Spain; 2 Eurecat Centre Tecnològic de Catalunya, Digital Health Unit, 08005 Barcelona, Spain; 3 Vall d’Hebron University Hospital, 08035 Barcelona, Spain; 4 ICREA, 08690 Barcelona, Spain

**Keywords:** lung cancer, tumour growth, uncertainty, deep learning

## Abstract

Predicting whether a lung nodule will grow, remain stable or regress over time, especially early in its follow-up, would help doctors prescribe personalized treatments and better surgical planning. However, the multifactorial nature of lung tumour progression hampers the identification of growth patterns. In this work, we propose a deep hierarchical generative and probabilistic network that, given an initial image of the nodule, predicts whether it will grow, quantifies its future size and provides its expected semantic appearance at a future time. Unlike previous solutions, our approach also estimates the uncertainty in the predictions from the intrinsic noise in medical images and the inter-observer variability in the annotations. The evaluation of this method on an independent test set reported a future tumour growth size mean absolute error of 1.74 mm, a nodule segmentation Dice’s coefficient of 78% and a tumour growth accuracy of 84% on predictions made up to 24 months ahead. Due to the lack of similar methods for providing future lung tumour growth predictions, along with their associated uncertainty, we adapted equivalent deterministic and alternative generative networks (i.e., probabilistic U-Net, Bayesian test dropout and Pix2Pix). Our method outperformed all these methods, corroborating the adequacy of our approach.

## 1. Introduction

Pulmonary tumour growth is one of the most important indicators when assessing lung cancer by computed tomography (CT) [1]. In the clinical practice, radiologists commonly assess tumour growth by measuring the longest diameter of all axial slices of the nodule on different CT scans taken at different time points [2]. Hence, unequivocal evidence of tumour growth (e.g., more than 2 mm in the longest diameter [3]) during follow-up is a clear symptom of malignancy, whereas a very rapid growth rate or stability over a 2-year time period are accepted indicators of benign nodules [4].

Being able to accurately anticipate whether suspicious pulmonary tumours will grow, remain stable or regress, especially when there are no previous imaging studies, would allow doctors to prescribe more personalized clinical management and better treatments for tumours. However, lung tumour progression is an unresolved prediction problem due to its heterogeneous (e.g., in size, texture and morphology) and multifactorial (e.g., genetic, environmental) nature [5]. In addition to this, tumour growth assessment is subject to intrinsic noise in medical imaging and inter- and intra-observer variability (up to 3 mm in diameter on spiculated nodules [6]) leading to questionable annotations to learn from. Traditionally, the tumour growth prediction problem has been addressed through sophisticated mathematical models, such as those based on the an reaction–diffusion equation [7]. Although these methods provide informative and explainable assumptions, they employ limited sets of handcrafted parameters (e.g., five in [8]), insufficient to model all the particularities of tumour growth patterns.

Nowadays, deep learning and in particular deep convolutional neural networks (CNN) have shown a great ability to automatically extract high-level representations from images [9]. This has enabled performance improvements over conventional approaches in various medical imaging problems [10], including lung cancer, such as nodule detection [11], malignancy classification [12] and nodule re-identification [13]. One of the earliest works using CNN for estimating future tumour growth was for pancreatic cancer [14]. The authors proposed the use of two (invasion/expansion) stream CNNs, relying on 2D patch images of the tumour for predicting future tumour segmentations. Best method performances achieved 86% of the Dice score. Those overcame the state of the art of conventional mathematical models [15] for that disease. However, the size of the test set was too small (10 cases) to extract robust conclusions. Furthermore, to make inferences, this network required multimodal images (i.e., dual phase contrast-enhanced CT and FDG-PET), which represented strong pre-conditions for the usability of the model. In other recent work [16], the authors proposed two 3D U-Net [17], one to predict the displacement field of the future tumour volume from a baseline and the other to refine the textural details, together with a temporal module to encourage time-aware representation learning. The network was trained with more than 300 pairs of 3D nodule-centred patches taken at two time points (baseline and follow-up studies). The gap in the model’s ability to provide future segmentations of the tumours (65% of the Dice score), the use of tailored criteria to determine nodule growth instead of conventional metrics (e.g., the longest diameter or double time volume) or not taking into account inter-observer variability, show the need to continue investigating more reliable and effective solutions. In another work [18], the authors proposed a network to combine 2D convolutional layers and gated recurrent units with an attention mechanism [19]. However, their goal was to predict spatial and temporal trajectories over a course of radiotherapy using a longitudinal magnetic resonance image (MRI) dataset, and the period of the predictions was weeks instead of months/years as in our case.

Recently, deep generative networks have been applied for the prediction of future tumour growth, motivated by their ability to generate new images. For instance, in [20] a 2D deep generative adversarial network (GAN) was proposed for discriminating between true tumour progression and pseudo-progression of glioblastoma multiform. In [21], the authors built a stacked 3D GAN for growth prediction of gliomas using temporal evolution of the tumour. Although high performances were reported (88% Dice score), the database was composed of only 18 subjects, in which all tumours always grew. In [22], the authors compared different GAN networks to predict the evolution of white matter hyperintensities. They also demonstrated the potential of using GANs in a semi-supervised scheme, improving results of a deterministic U-ResNet [23]. Despite the satisfactory performances obtained with GANs, this type of network suffers from mode collapse [24]; that is, they hardly generate correct representations of the probability output distribution, so they may not be adequate to model uncertainty. Alternatively to the use of GAN-based architectures, few works have also applied auto-encoders and their variants for tumour/disease growth prediction. In [25], the authors applied a variational auto-encoder (VAE) for progression of Alzheimer’s disease from structural magnetic resonance imaging (MRI). Their experiments demonstrated that VAE outperforms conventional CNNs in doubtful cases, since it acts as a soft classifier learning a Gaussian distribution. However, CNNs provided better overall performance. In another study [26], the authors conditioned a deep auto-encoder on fixed characteristics, such as age and diagnosis, to generate sequences of 3D MRI for Alzheimer’s disease progression. Although results outperformed previous 2D versions, some artefacts and false structures were noted on the generated images.

Beyond the complexity of the problem, different factors undermine the trustworthiness of existing solutions, such as the variability in the ground truth (multiple tumour growth hypothesis by different radiologists) or the inherent uncertainty in the images due to resolution, contrast and noise limitations, which in turn also lead to variability in the outputs produced by different radiologists [6]. Uncertainty estimation in deep neural networks has been widely investigated for medical image tasks. For instance, in segmentation of multiple sclerosis lesions, some work [27] showed that by filtering out predictions with high uncertainty, the models improved lesion detection accuracy. For brain tumour segmentation, other work [28] demonstrated that MCDNs can be calibrated to provide meaningful error bars overestimates of tumour volumes. Unfortunately, few works have modelled uncertainty for future tumour growth estimation. In [29], a deep probabilistic generative model (sPUNet) [30] was used to model glioma growth for radiotherapy treatment planning. The model, based on a combination of a U-Net [31] and a CVAE [32], was able to generate multiple future tumour segmentation modes on a given input. Although they demonstrated the potential of providing multiple views over a single solution, they did not report nodule growth performances. Recently, an updated version of this method was presented [33] to improve some limitations of the previous approach, such as the need for fixed time intervals between observations.

In contrast, given the multifactorial and complex nature of estimating future tumour growth, uncertainty in the prediction was addressed in any of the aforementioned studies. Therefore, further efforts are required to build accurate solutions that predict future tumour growth and quantify the uncertainty arising from the aforementioned factors in order to avoid possible misinterpretations and misdiagnosis. Along with recent interest in prediction of future tumour growth and uncertainty with deep learning, this work aims to take a step forward in these promising research directions. In particular, we propose a probabilistic generative network able to predict, given a single time-point image of the lung nodule, multiple consistent structured output representations. To do this, the network learns to model the multimodal posterior distribution of future lung tumour segmentations, given by multiple expert opinions, by using variational inference and injecting the posterior latent features. Eventually, by applying Monte Carlo sampling on the outputs of the trained network, we estimate the expected tumour growth mean and the uncertainty associated with the prediction.

To the best of our knowledge, the following points are the novel contributions of this work in the field of tumour growth prediction:

- This is the first time that future pulmonary nodule growth is estimated using deep learning and nodule diameter annotations from multiple experts.
- This is the first time that uncertainty is reported using a deep learning approach to predict lung nodule growth.
- The present approach, based on the hierarchical probabilistic segmentation framework, integrates a novel loss function, a new post-processing to obtain lung nodule growth prediction, quantification and visualization with the corresponding associated uncertainty and a new evaluation metric for measuring the generation ability of the network.

## 2. Materials and Methods

**Figure 1:**
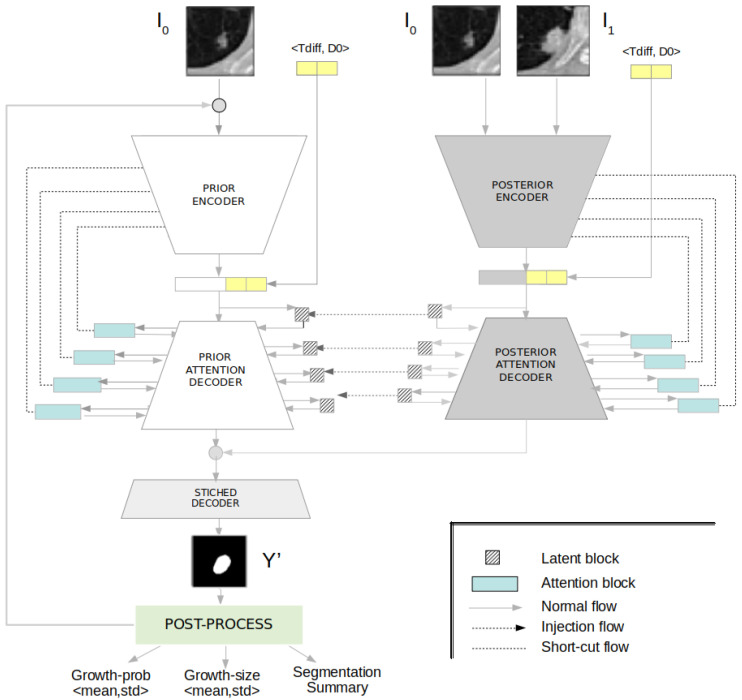
Overview of the U-HPNet composed of two subnets. The prior (on the left), the posterior (on the right), and the post-process module (at the bottom) are aimed at reporting the estimated future growth prediction, size and appearance with the associated uncertainty.

We present an uncertainty-aware hierarchical probabilistic network (U-HPNet) to predict future lung tumour growth given a baseline image of the tumour I_0_ at time T_0_ (Figure 1). This method, unlike previous solutions, takes into account the inherent uncertainty present in lung CT scans. To do this, the network approximates the posterior probabilistic distribution of tumour growth, using as ground truth multiple expert opinions. For this, the network relies on an existing hierarchical and probabilistic framework [34,35] (HPU), further details in Section S1 of the Supplementary Materials. Hence, we adapted the HPU to generate plausible future tumour segmentations $Y^{\prime }$ in its follow-up at time T_1_. In addition, through a Monte-Carlo sampling post-process on the outputs of the network, we derived the expected tumour growth and its associated uncertainty.

In the next sections, we describe the input, output and architecture of the network (Section 2.1). We detail its training and inference process (Section 2.2). We explain the post-process (Section 2.3). We present some alternative solutions (Section 2.4), and finally, we specify the experimental setup for training and evaluating the network (Section 2.5).

### 2.1. Architecture

The U-HPNet, as the HPU framework, is composed of two subnetworks: prior and posterior (Figure 1). The prior subnet was created to model the prior distribution of future tumour segmentations given the baseline image of the nodule. Thus, we configured its input with the initial image of the nodule I_0_ at time T_0_. The posterior subnet was created to model the posterior distribution of future tumour segmentations. Hence, we configured its input with the images of the nodule at both time points (i.e., I_0_ and I_1_).

The input images of both subnets are 2D axial patches, centred on the nodule, and with a size of 32 × 32 pixels. The alignment of the nodules to their centroid facilitated the prediction of their evolution. However, using smaller patches was meaningless due to the size of the nodules, and adopting larger patches (e.g., 64 × 64) experimentally did not report any performance gain. Adding other images into the input of the prior (e.g., segmentation of the nodule at T_0_) and/or the posterior (e.g., segmentation of the nodule at T_1_) subnets were explored, but they did not report better performances. To favour the recognition of growth patterns between tumour images, both subnets were configured to also receive as input the time difference T_diff_ at which to predict the future nodule segmentation. This parameter is encoded as an ordinal value representing the main elapsed time defined by radiological guidelines (i.e., 6, 12, 24 or more months) [3]. Additionally, we included another input parameter to these subnets, the diameter (D0) of the nodule at T_0_. This feature was added because after experimentation it reported a slight performance gain, and it was always available at runtime. Both parameters (T_diff_, D0), were normalized between 0 and 1, and concatenated with the encoder output.

**Figure 2:**
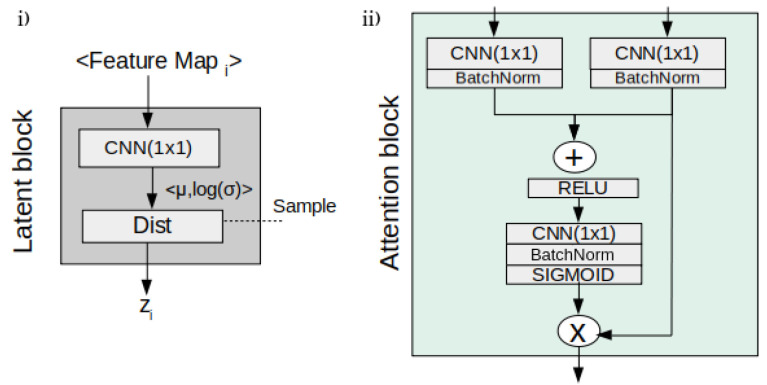
Details of the latent (**i**) and the attention (**ii**) blocks of the U-HPNet.

Regarding the core architecture of the U-HPNet, as in the original HPU framework, both subnets were implemented with 2D U-ResNets adapted to the proposed input/output size (32 × 32) and the additional described parameters. In addition, up to 4 prior/posterior latent blocks (Figure 2) were interleaved in the decoder of the subnets. These 4 latent blocks encode the input feature activations into probabilistic latent distributions, from which the latent feature vectors (z_i_) of 1, 4, 16 and 64 dimensions are sampled, respectively. We explored using less latent blocks, but we did not obtain better performance. Adding more latent blocks was not possible due to the limited size of the activation maps at the fourth latent block.

Additionally, we incorporated in the decoder of both subnets a soft attention mechanism (Figure 2) with the intention of detecting small and minor changes in the structure of the nodule images. To do this, we added attention gates [19] before the concatenation operation to merge only relevant activations.

**Figure 3:**
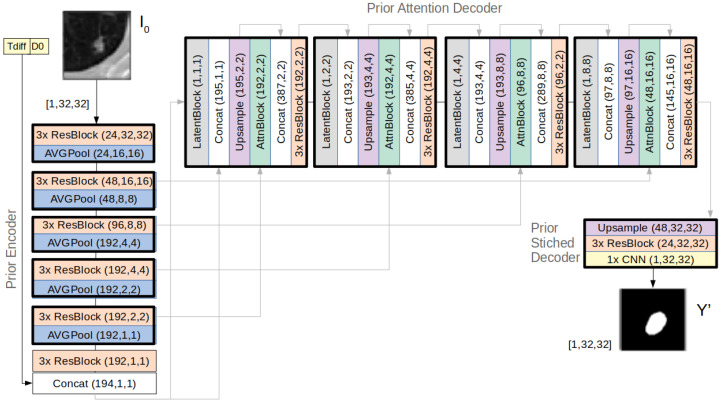
Architecture details of the prior subnet of the U-HPNet (the posterior subnet follows the same architecture, but also receives I_1_ as input).

Figure 3 provides further details of the architecture of the prior subnet, where it is possible to observe the components of the latent and attention blocks and how they were integrated in the decoder.

### 2.2. Inference and Learning Process

The inference process of the U-HPNet consists of forward-passing the input information of the tumour at T_0_ (i.e., $I_{0},\;T_{\mathrm{diff}},\;D0$) through the prior subnet (Figure 3) to obtain $Y^{\prime }$, a future segmentation of the tumour at T_1_. For this, along the decoder of the prior subnet, feature activation maps are concatenated with vectors z_i_ (i ≤ L, being L = 4 the number of latent hierarchies), obtained from sampling the different latent distributions interleaved in the decoder. With just another forward-pass of the same input through the prior subnet, and sampling into each of the latent distributions, the network is able to generate a new sample.

The training process of the U-HPNet aims at minimizing the loss of the reconstructed image segmentation (i.e., the output of the prior subnet using or injecting the latent feature vectors z_i_ from the posterior subnet), while pulling the variational posterior distribution $Q(.|I_{0},\;I_{1},\;T_{\mathrm{diff}},\;D0)$ of image segmentations, defined by the posterior subnet, towards the prior distribution $P(.|I_{0},\;T_{\mathrm{diff}},\;D0)$ of the reconstructed image segmentations. Hence, during the training, the network minimizes the reconstruction loss, $L_{\mathrm{rec}}$, between the estimated $Y^{\prime }$ and real *Y* future segmentation of the lung nodule at T_1_ and the KL divergence D_KL_ between the variational posterior and the prior distributions.

For the reconstruction loss, we propose a new loss function tailored to the problem to solve. Thus, we formulate the reconstruction loss as the sum of the L1 distance between the longest diameter $D1^{\prime }$ obtained from the predicted future tumour segmentation and the ground truth tumour diameter D1 and the intersection over union (IoU) between the predicted $Y^{\prime }$ and ground truth *Y* tumour segmentation. This loss experimentally provided better results than the cross entropy (even with online negative mining) as proposed originally in the HPU framework. This can be explained because the L1 loss allows prioritizing the fidelity to the ground truth diameter, and the IoU loss is a good metric for learning on imbalanced data conditions [36]. Additionally, a weighting (γ) factor was used on the combined loss to balance both terms:

$$L_{\mathrm{rec}}=L_{\mathrm{IoU}}\left(Y,Y^{\prime }\right)+\gamma L_{L1}\left(D1,\;D1^{\prime }\right).$$

Thus, the overall loss of the U-HPNet is as follows:

$$L_{\mathrm{ELBO}}=L_{\mathrm{rec}}+\beta (\sum_{i=0}^{L}E_{z_{<i}∼Q}D_{\text{KL}}(q\left(z_{i}|z_{<i},\;I_{0},\;I_{1},\;T_{\mathrm{diff}},D0\right)\left|\right|$$

$$p(z_{i}|z_{<i},I_{0},T_{\mathrm{diff}},D0)))$$

In Section 3.2.3, we include an ablation study on the different components of both the architecture and the loss function of the U-HPNet.

### 2.3. Uncertainty in the Predictions

To estimate future tumour growth and its associated uncertainty, we incorporate a post-processing module in the U-HPNet that applies Monte Carlo sampling by running the network *K* times (*K* = 1000) with the same input image. As a result, we obtain *K* nodule segmentations. For each predicted segmentation *k*, we extract its longest diameter $D1_{k}^{\prime }$, using conventional image processing libraries. With the vector of *K* nodule diameters, we compute the vector of predicted nodule growths, $\Delta \in R^{K}$, by subtracting the input nodule diameter size D0 to each of the predicted diameters $D1_{k}^{\prime }$. From Δ, we compute its mean $\bar{\Delta }$ and standard deviation $s_{\Delta }$ as measures of nodule growth size and its associated uncertainty.

In addition, we compute the probability that the nodule growth is at least 2 mm (threshold recommended in clinical guidelines for tumour growth [3]). For this, for each of the K nodule growth estimates, we used the logistic function:

$$F_{k}=1/(1+e^{-\Delta_{k}+2}).$$

From the resulting *K*-length vector of probabilities, $F\in R^{K}$, we consider the mean $\bar{F}$ and the standard deviation $s_{F}$ as the estimated nodule growth probability and its associated uncertainty. Finally, the post-processing module also outputs two images, both corresponding to the predicted future tumour appearance (at T_1_). In particular, and inspired by [37], one of the images is the per-pixel mean of all *K* predicted segmentations and the other the per-pixel standard deviation.

### 2.4. Comparison with Related Works

**Figure 4:**
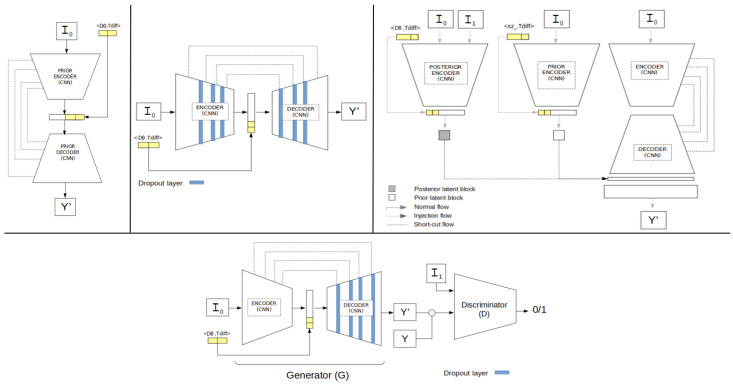
Four alternative network architectures adapted for lung nodule growth estimation. At the top, we have the U-ResNet(U-Net), the generative Bayesian dropout (BAYES_TD), and the probabilistic U-Net (SPU). Below, we find the Pix2Pix cGAN network (GAN-P2P).

Since we did not find similar approaches providing future lung tumour growth prediction (using the longest nodule diameter) along with their associated uncertainty, we implemented and adapted 4 different alternative deep neural networks (Figure 4) to compare the performance of our method: one deterministic (U-Net) [31] and 3 generative networks, i.e., the probabilistic U-Net (SPU) [30], the Bayesian dropout (BAYES_TD) [37] and the Pix2Pix (GAN-P2P) [38].

To allow a fair comparison, all these networks had the same U-ResNet backbone proposed for the U-HPNet, with the same number of layers and filters. Moreover, these networks had the same input (I_0_, D0 and T_diff_) and output as the U-HPNet (i.e., an estimated future segmentation of the nodule), with the same post-processing (only for the non-deterministic networks). Further technical details can be found in Section S2 of the Supplementary Materials.

### 2.5. Experimental Setup

#### 2.5.1. Dataset

**Figure 5:**
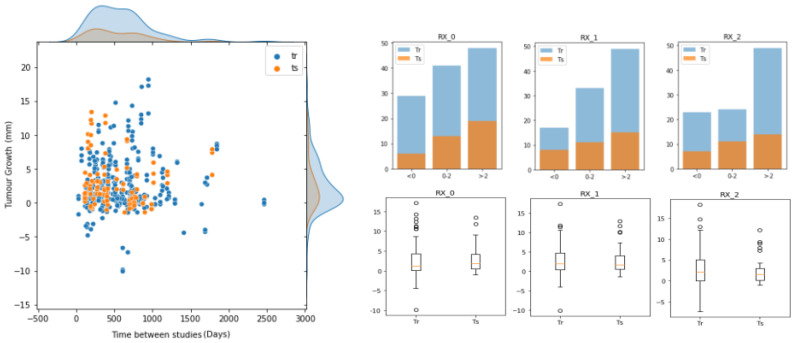
Description of the training and test set cohorts in terms of tumour growth size (in mm), time between studies (days) and the annotators (RX-0,1,2).

To build, evaluate and compare our approach for predicting future lung nodule growth, we used a cohort of 161 patients with incidentally discovered lung nodules (Figure 5), with two thoracic CT scans per patient. Inclusion criteria were patients without a previous neoplasia, with a confirmed diagnosis, and with visible nodules (≥5 mm) in at least two consecutive CT scans.

The most relevant pulmonary nodule in each patient was located in the three spatial axes by two different specialists at each time point and quantified by up to 3 different radiologists (RX0, RX1, and RX2). The annotators reported the diameter size of the nodules (D0, D1) at the two different time-point ($T_{0}$, $T_{1}$) studies. From here, we computed the ground truth of tumour growth by subtracting the diameters (D1−D0). The tumour growth mean in the dataset was 2.52 ± 3.85 mm for RX0, 2.76 ± 3.63 mm for RX1, and 2.68 ± 4.01 mm for RX2. The inter-observer mean absolute difference was 1.55 mm, whereas the inter-observer mean standard deviation was 0.97 mm (both metrics were computed pair-wise). The time interval between baseline and follow-up CT studies ranged from 32 to 2464 days. Tumour segmentations were obtained in a semi-automatic way, being visually verified and curated with the annotations provided by each of the radiologists (that is, location of the centroid, diameter and growth of the tumours). For training and evaluation purposes, the dataset (Figure 5) was randomly divided into training (70%) and test (30%) sets. In this process, we ensured that all entries of the same nodule were in the same set in order to avoid data leakage between partitions. Therefore, the training set was composed of 313 (122 unique) nodule growth annotations from up to three different radiologists (118 for RX0, 99 for RX1 and 96 for RX2), whereas the test set was formed by 104 (39 unique) nodule growth annotations (38 for RX0, 34 for RX1 and 32 for RX2). Hence, for each data entry (i.e., nodule growth annotation) of these partitions, we had 2 nodule images (at T_0_ and T_1_), 2 nodule segmentations (at T_0_ and T_1_), a growth label (indicating whether it grew (1) or not (0)) and a growth size (in mm) corresponding to a particular radiologist.

#### 2.5.2. Tumour Growth Assessment

In addition to the annotations provided by the three radiologists (RX0, RX1 and RX2), we derived two more ground truths to provide further insights in the evaluation of the methods: the mean of the radiologist annotations and the radiologist annotations that stand closest to our predictions. To identify the closest radiologist for nodule growth probability and tumour growth size, we selected the radiologist annotation with the minimum Mahalanobis distance between the radiologist’s measure (of the growth size) and the predicted growth vector Δ. To select the closest radiologist in terms of future tumour segmentation, we searched for the radiologist with the highest average Dice score between the semi-automatic segmentation generated by the radiologist and the generated segmentation samples of the network.

To assess the performance of the predictive models, we proposed two different evaluation scenarios (both taking into account the performance of the network in terms of nodule growth probability, size and segmentation). The first scenario evaluates the mean of the estimated probabilistic distribution. Since this implies providing a single value per tumour, we used conventional metrics such as precision (Prec), recall (Rec), and balanced accuracy (Bacc) for nodule growth probability $\bar{F}$, mean absolute error (MAE) for growth size $\bar{\Delta }$, and Dice for segmentation fidelity. The second scenario evaluates how well the estimated output distribution fits with the ground truth distribution. Thus, we proposed 2 complementary metrics:

- *Generalized energy distance* (GED) [39], which reports the segmentation performance in terms of the variability in the ground truth as in the generated samples of the network.
  $$D_{GED}^{2}=\frac{2}{nm}\sum_{i=1}^{n}\sum_{j=1}^{m}d\left(Y_{i}^{\prime },Y_{j}\right)-\frac{1}{n^{2}}\sum_{i=1}^{n}\sum_{j=1}^{n}d\left(Y_{i}^{\prime },Y_{j}^{\prime }\right)-\frac{1}{m^{2}}\sum_{i=1}^{m}\sum_{j=1}^{m}d\left(Y_{i},Y_{j}\right).$$
  where *m* and *n* are the number of generated and ground truth segmentations, $Y_{i}^{\prime }$ and $Y_{j}$ are a predicted and ground truth tumour segmentations, and *d* is the distance obtained using the 1-IoU metric. The closer the GED is to 0 the better.
- *Balanced accuracy at 2 standard deviations* (Bacc_2std) reports the balanced accuracy between the ground truth growth sizes and the predicted tumour growth sizes at 2 standard deviations away from the estimated growth size means (i.e., 1 if ground truth growth size > 2 mm and $(\bar{\Delta }\pm 2s_{\Delta })>$ 2 mm). Thus, we re-defined a true positive as when the ground truth growth size and the lower value of the predicted interval were above 2 mm. A false negative was when the ground truth growth size was above 2 mm, but the lower value of the interval was not. A true negative was when the ground truth size and the upper value of the interval were less or equal to 2 mm. False positive was when the ground truth size was less or equal to 2 mm, but the upper value of the interval was not.

#### 2.5.3. Model Configuration

The proposed network was configured with a learning rate of 1e-4, a batch size of 8, γ to 1/8, β to 1, 200 epochs and Adam [40] as the optimization algorithm. These parameters were obtained by a 5-fold cross-validation on the training dataset, applying conventional data augmentation techniques (i.e., rotation, translation) on the images. The alternative methods, implemented to compare our approach, were configured with the same data augmentation and optimization parameters. All methods were tested on the independent test set.

## 3. Results

### 3.1. Qualitative Results

**Figure 6:**
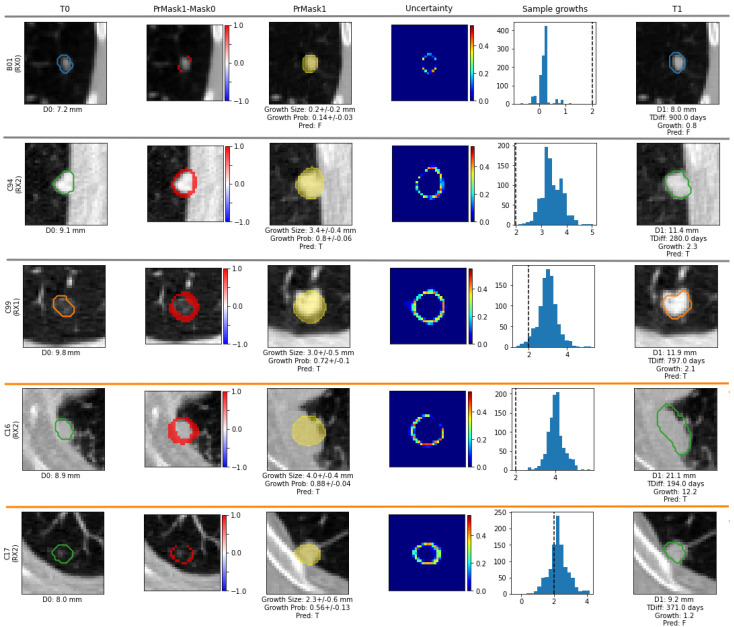
Visualization results of five different test cases. The first column is the initial nodule at T_0_ overlapped with the segmentation of a radiologist. The second shows, overlapped with the nodule at T_0_, the difference between the ground truth segmentation at T_0_ and the predicted mean segmentation at T_1_. The third provides, overlapped with the nodule at T_1_, the predicted tumour mean segmentation. The fourth is the estimated uncertainty probability map with the per-pixel standard deviation. The fifth shows the histogram of the (K = 1000) estimated tumour diameter growths (dashed line is the 2 mm tumour growth threshold). The last shows the nodule at T_1_ overlapped with the segmentation of the radiologist at T_1_.

Following, we present some qualitative results (five cases) obtained from the U-HPNet, using as input nodule images from the test set (Figure 6).

In the first case, B01, the radiologist (RX0) did not detect any relevant tumour growth (diameter difference of 0.8 mm, ≤2 mm). Our method correctly predicted the existence of no growth, reporting a low tumour growth probability of 0.14 ± 0.03 and a tumour growth size of 0.2 mm. In the second tumour case, C94, which was attached to the chest wall, the radiologist (RX2) detected a relevant tumour growth (diameter difference of 2.3 mm, >2 mm). Our method correctly predicted tumour growth reporting a high growth probability of 0.8 ± 0.06 and tumour growth size of 3.4 mm. The third case, C99, shows a part-solid tumour with a diameter growth of 2.1 mm by RX1. Our method correctly predicted tumour growth, although with a probability of 0.72 (with a 10% of uncertainty) and a tumour growth size of 3.0 mm. For the case C16, our model correctly predicted tumour growth. However, the radiologist (RX2) indicated a high tumour increase of 12.2 mm,, whereas the network only detected 4.0 ± 0.4 mm with a probability of 0.88 ± 0.04. If we observe the estimated segmentation mean image of the nodule, we see that the network missed detecting the longitudinal growth direction of the tumour, probably because of the lack of similar cases in the training set. In the last case, C17, the model incorrectly predicted tumour growth with a diameter difference of 2.3 ± 0.6 mm, whereas the radiologist (RX2) reported no relevant tumour growth (diameter difference of 1.2 mm). Nevertheless, the network provided a growth probability close to 0.5 with an uncertainty of 0.13, which implies that the network was not confident on the nodule predictions. Looking at the ground truth provided for this case (first column of the figure), this mistake could be due to a diameter overestimation at T_0_ by the radiologist RX2. Further cases are provided in Section S3 of the Supplementary Materials.

### 3.2. Quantitative Results

#### 3.2.1. Tumour Growth Estimated Means

Table 1 shows the test performances of the U-HPNet obtained using the predicted means and the annotations from three different radiologists (RX0, RX1 and RX2), their mean and the radiologists’ annotations closest to our predictions (closest). More details on the description of the metrics and their units can be found in Section 2.5.2.

**Figure 7:**
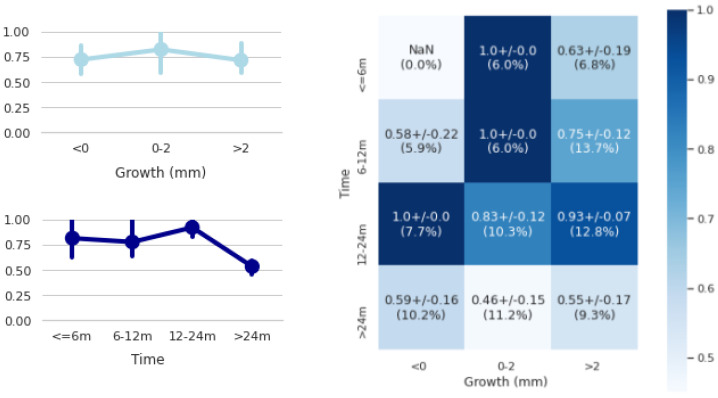
Tumour growth performances of the U-HPNet stratified by nodule growth sizes (top-left), by time to predict (bottom-left) and by both factors (on the right) using the closest ground truth criterion. In each cell of the confusion matrix, we show the accuracy and, in parentheses, the percentage of cases of the test set. The NaN means that there were no data in the test set for this combination.

According to the best results (i.e., with the closest criterion), our method was able to report positive performances, such as 0.74 of balanced accuracy for future nodule growth probability, 1.74 mm of MAE for future tumour growth size (close to the 1.55 mm of inter-observer mean absolute difference) and 0.78 of segmentation Dice score in its follow-up. Regarding the tumour growth probability performance, we broke it down by the time to predict and the nodule growth size (Figure 7). From this figure, we can observe that the best performances were usually obtained when predicting in the range of 12 to 24 months (92% accuracy) and when the nodules had a growth between 0 and 2 mm (82.3% accuracy). The worst performances were obtained when predicting above 24 months (53.3% accuracy). However, the method reports an accuracy of 84% when the most distant predictions (greater than 24 months) are excluded.

#### 3.2.2. Tumour Growth Estimated Distribution

Complementary, we evaluated the ability of the network to generate accurate and varied future tumour segmentations (Table 2). To do this, we computed the GED and Bacc_2std metrics. From the GED results, we remark that a high segmentation agreement (i.e., 0.14 of 1-IoU) was found between ground truths (YY). This may explain why a small variability (i.e., 0.04 of 1-IoU) was also found between predicted segmentations (Y′Y′). From the reported Bacc_2std results, we saw that at this limit (i.e., two standard deviations), our approach was still able to correctly predict tumour growth in 57% of the test cases with the closest radiologist ground truth.

#### 3.2.3. U-Hpnet Ablation Study

An ablation study was conducted to isolate the effects of the different components of the U-HPNet using the radiologists’ annotations closest to our predictions. Table 3 shows the different network setups evaluated and their acronym for better identification.

Table 4 shows the performances obtained for tumour growth prediction, size and segmentation for each of the network setups using their estimated means. The configuration using binary cross entropy (BD0) was clearly outperformed by those using IoU. Particularly, a rise of 0.08 in Bacc and an improvement of 0.19 mm in MAE was obtained with the U-HPNet, and an increase of 0.07 in the Dice score was reached with the IDAOD0. In addition, the networks using attention (i.e., U-HPNet and IDAOD0) obtained the best performances in terms of MAE, in particular the U-HPNet obtained the lowest value with 1.74 mm. Regarding the Dice score, all networks using IoU loss obtained performances above 0.78, although the IDAOD0 with 0.81 was the one with the highest performance.

### 3.3. Comparison with Other Networks

Since we did not find similar approaches using the longest diameter to provide lung tumour growth prediction along with its associated uncertainty, we adapted four different alternative deep neural networks: one deterministic (U-Net) [31] and three generative networks, i.e., the probabilistic U-Net (SPU) [30], the Bayesian dropout (BAYES_TD) [37] and the Pix2Pix (GAN-P2P) [38].

Table 5 shows the test performances obtained for the proposed networks using as ground truth the radiologists’ annotations closest to our predictions. For the deterministic network, we used the predicted value and for the generative methods, the mean of the output distribution.

From the four alternative methods, the SPU obtained the best Bacc score with 0.73 and MAE with 2.14 mm. In contrast, the BAYES_TD method obtained the best Dice score with 0.78. If we compare these results with the U-HPNet, none of them could outperform their results in terms of prediction, size or segmentation.

In Table 6, we summarize the performances regarding the generative ability of the proposed methods. The alternative approach with the best Bacc_2std was BAYES_TD with 0.46 and with the best GED was SPU with 0.23. Thus, the output distributions of these methods reported slightly better segmentation variability (lower GED), although worse accuracy (lower Bacc_2std) than the U-HPNet.

## 4. Discussion

Having accurate and reliable models for predicting the evolution of lung tumours in early stages of their follow-up would help to better understand the risk of the disease and provide personalized treatments [1]. Thus, we presented a method that predicts, given a single image of the pulmonary nodule, its future progression at a given time. To this end, and in accordance with current clinical practice, our method predicts growth when there is a substantial increase (i.e., more than 2 mm) in the largest diameter obtained from all the axial slices of the pulmonary nodule [3]. Although this criterion is commonly used for its simplicity and applicability, it entails significant inter-observer [6] variability that may impact the reliability of the predictive models. Other inter-related factors also have a direct impact on the trustworthiness of the estimator, such as the fact that lung nodules are not always clearly visible due to the noise inherent in medical imaging. Particularly, the aleatory uncertainty is related to the intrinsic variation of the system caused by model inputs, which can lead to an unpredictable variation in the outcomes [41]. In the case of predicting the growth of lung tumours, the uncertainty caused by the images entails a relevant variability between the outputs provided by radiologists [6]. Therefore, modelling this variability among radiologists (the ground truth) can be seen as a way to approximate the uncertainty in the data, as proposed in [42].

To estimate lung tumour growth along with its uncertainty, we collected a longitudinal dataset with more than 160 selected pulmonary tumours with two CT images per case (taken at different time points), labelled by up to three different radiologists. To model these data, we opted for a generative deep learning approach, as opposed to the deterministic approaches used to date [16,18] for lung tumour growth prediction. The suitability of the generative approach was already proved in [29], where they modelled glioma tumour growth using an early probabilistic and generative framework [30] to estimate the tumour growth output distribution. Nonetheless, tumour growth prediction was not quantified, model uncertainty was not reported, and multiple observer variability was not contemplated. To address the aforementioned aspects, we relied on a recent hierarchical generative and probabilistic framework [34,35] to estimate the output distribution of the future lung tumour appearance (at T_1_) conditioned on the previous image of the nodule (at T_0_). Our method (U-HPNet) extended this framework with the following modifications. First, we adapted the inputs of the network and added two new features to extract additional patterns from the tumour images: the time to predict and the diameter of the nodule (at T_0_). Second, we integrated an attention mechanism [19] in the decoder part of the network to boost its performance. Third, we proposed a new reconstruction loss function composed of the IoU and the L1 distance to provide more accurate segmentation and diameter estimations. Finally, we created a new post-processing module that applies Monte Carlo sampling to estimate the mean and standard deviation of the tumour growth prediction, diameter growth and segmentation of a given nodule at an specific time.

We evaluated the U-HPNet using the annotations provided by three different radiologists, but also with their average and the radiologists’ annotations closest to our estimates, to provide a more complete assessment of our approach and to detect possible divergence between the experts.

Regarding the evaluation of our approach using the predicted means, the best results were obtained using the radiologists’ annotations closest to our predictions. This ground truth criterion always reports real radiological annotations (specifically, the closest ones to our predictions); therefore, since we take all radiologists’ opinions equally, this criterion is comparable to any of the three radiological criteria available in the study. In particular, we achieved 74% of tumour growth balanced accuracy (Bacc), 1.74 mm of diameter mean absolute error (MAE) and 78% of the Dice score (Table 1). However, 84% accuracy was achieved in predicting future tumour growth when predictions greater than 24 months were not taken into account (Figure 7). Similar recent work in the literature [16] reported higher balanced accuracy scores (86%) but much lower segmentation Dice scores (64%) than us. Results, however, are not fully comparable since both networks used different in-house cohorts, with different tumour case complexities, and both defined tumour progression differently; theirs relied on a tailored volumetric threshold and ours on the diameter growth convention established in radiological guidelines ([3]).

We also evaluated the ability of the network to produce consistent samples (see Section 2.5.2) matching with the closest radiologists’ criterion. Regarding the GED performance (Table 2), we observed that the network obtained 23% of segmentation variability between predicted and ground truths (Y’Y), being not far from 14% of inter-observer variability (YY). In addition, the network showed a relatively small variability of 4% between the generated sample segmentations (Y’Y’). This may indicate that during training the network preferred to concentrate the predictions around the mean rather than predict highly disperse values in order to optimize performance. With respect to the Bacc score (Bcc_2std), we obtained a 17% lower performance than using the predicted mean of tumour growth. This reflects that our approach still has room for improvement to generate samples that bring the tumour growth size mean closer to the radiologists’ ground truths. To accomplish this, different solutions could be applied to improve the network performance, such as acquiring more tumour cases, especially in those cases where the method was not as accurate (>24 months), using more aggressive data augmentation techniques, or analysing volume images rather than single slices, to extract better predictive features.

For a better understanding of the effects of the design decisions of the U-HPNet, we provided an ablation study with different network configurations using the closest radiologists’ criterion (Table 3). From this analysis, we learned that the largest improvement was achieved replacing binary cross entropy (BCE) by IoU in the reconstruction loss. This can be observed by comparing BD0 and ID0 networks. Specifically, the Bacc increased approximately 6%, MAE decreased almost 0.13 mm, and the Dice improved to nearly 5%. Different reasons may explain the suitability of using IoU for this problem. First, this loss is robust to data unbalance. Second, IoU had values with similar magnitude to the KL-divergence distance, allowing a better optimization of the network than using BCE. A second network configuration (IDD0) allowed improving previous performance limitations. In particular, this network incorporated the L1 distance between the predicted and ground truth diameters in the reconstruction loss together with IoU. Results showed that the IDD0 network increased its growth prediction performance (1% in Bacc) and segmentation ability (1% in the Dice and GED) slightly, despite a slight decrease of performance in diameter growth prediction (0.04 mm in MAE). Adding attention (IDAOD0 network) in the decoder part of the subnetworks outperformed the IDD0 precisely, reducing 0.05 mm in MAE and increasing 1% its Dice score. A final comparison was performed between IDAOD0 and U-HPNet to obtain the importance of adding nodule diameter (at T_0_) in the input of the U-HPNet. Based on the results, using this feature, we reduced 0.05 mm of MAE, and we increased 1% of Bacc.

Due to the lack of studies addressing the prediction and uncertainty of future lung tumour growth through the longest axial diameter of the nodule, we built different alternative networks to allow their comparison in the collected dataset. In particular, we proposed a deterministic (U-Net), and three different generative networks: Bayesian dropout (BAYES_TD), probabilistic U-Net (SPU), Pix2Pix GAN (P2P_GAN). The comparison was performed using the closest radiologists’ criterion. Results from Table 5 and Table 6 showed that using the estimated sample means, the generative approaches outperformed the performances reported by the deterministic network (U-Net). This result consolidates the suitability of the generative approach for this type of problem. In addition, among all generative methods, the U-HPNet obtained the best performance metrics using the estimated means (i.e., in tumour growth prediction, size and segmentation). Regarding the metrics measuring the generative ability of the networks, the U-HPNet obtained the best Bacc_2std. In contrast, the SPU reported a large sample variability in tumour growth size as shown by the lowest GED, but one of the lowest performances in Bacc_2std. The GAN-P2P, similar to the SPU, obtained low GED but poor Bacc_2std due to high variability in the sample distribution of tumour growth size. The BAYES_TD, in contrast, obtained lower variability in tumour growth size, achieving better GED than the U-HPNet but lower Bacc_2std. Interestingly, BAYES_TD and U-HPNet networks reported rather similar generative performances, despite employing two different ways to generate samples (by weight randomization and by randomly selecting a vector in the latent space). Thus, combining both approaches could help disentangle different types of uncertainties (as in [42] for tumour segmentation) and disclose a potential increase in the performance of the network.

Our method still has a number of limitations. First, the number of tumour cases analysed in this study was low, which clearly impacted the reported performances of our approach due to its data-eager nature. However, these data were clinically validated and selected by different radiologists according to their relevance and interest. Although there are different initiatives that aim at screening large populations at risk (e.g., NLST National Lung Screening Trial. https://www.cancer.gov/types/lung/research/nlst (accessed on 15 September 2022)), the clinical management of these assets differs from non-screening cohorts (e.g., image studies are taken routinely every year), and they usually come only with annotations (i.e., diameter) provided by a single expert due to the size of the cohort. Second, segmentations were generated semi-automatically according to the original diameter, growth and centroid annotations with a final visual expert validation. However, we believe using manual expert segmentations could make our method more precise, especially in the contour of the tumours. Third, our method relied on a single axial slice of the tumour to predict tumour growth. However, tumour growth is a tridimensional biological process; hence, using volumetric images may allow capturing further relevant features and patterns to better explain tumour progression. Nevertheless, clinical practice still relies on manual 2D measurements (e.g., the longest axial nodule diameter).

Beyond this work, further efforts in fine-tuning the proposed approaches, such as exploring different number of layers, latent variables, loss weights factors and other optimization parameters, are required. In addition, future extensions to this article are suggested, such as exploring a 3D version of the network, deepening in the uncertainty ability of the network (e.g., reporting epistemic uncertainty), evaluating its integration with Bayesian dropout or adversarial learning and incorporating the newest advances in deep learning to extract better spatial and temporal features from the tumours.

## 5. Conclusions

In this article, we addressed future lung tumour growth prediction as a generative multimodal output problem as opposed to existing deterministic solutions that provide a single output. Several reasons motivated our decision, such as the complexity of the problem, the inter-observer variability, and the importance of estimating uncertainty in medical settings. To this end, we proposed a deep hierarchical generative and probabilistic network that, given a single image of the nodule, predicts and quantifies future tumour growth and visualizes the semantic appearance of the tumour along with its associated uncertainty. The network was trained and evaluated in a longitudinal cohort, and its results in predicting tumour growth, future size and expected segmentation outperformed alternative solutions based on current approaches.

## Figures and Tables

**Table 1:** Nodule growth prediction performances obtained by the U-HPNet using estimated means. The down arrow means the lower the score the better, and the best results are in bold.

RX	Bacc	Prec	Rec	MAE ↓	Dice
RX0	0.49 ± 0.07	0.46 ± 0.11	0.42 ± 0.11	2.99 ± 0.42	0.74 ± 0.02
RX1	0.68 ± 0.08	0.63 ± 0.13	0.64 ± 0.13	2.52 ± 0.45	0.77 ± 0.02
RX2	0.67 ± 0.09	0.58 ± 0.15	0.59 ± 0.15	2.62 ± 0.41	0.75 ± 0.02
Mean	0.55 ± 0.04	0.50 ± 0.07	0.47 ± 0.07	2.83 ± 0.23	0.76 ± 0.02
Closest	**0.74** ± **0.07**	**0.65** ± **0.12**	**0.71** ± **0.12**	**1.74** ± **0.34**	**0.78** ± **0.02**

**Table 2:** GED nodule segmentation performance of the U-HPNet. Each score reports 1-IoU.

GED ↓	2 × (Y′Y)	Y′Y′	YY
0.29 ± 0.04	0.48 ± 0.04	0.04 ± 0.01	0.14 ± 0.01

**Table 3:** Different network setups of the U-HPNet configured in the ablation study.

Acronym	Lrec	Attention	D0
BD0	BCE	✗	✓
ID0	IoU	✗	✓
IDD0	IoU+L1	✗	✓
IDAOD0	IoU+L1	✓	✗
U-HPNet	IoU+L1	✓	✓

**Table 4:** Performance comparison between the different U-HPNet setups.

	Prediction	Size ↓	Segmentation
	(Bacc)	(MAE)	(Dice)
BD0	0.66 ± 0.09	1.93 ± 0.35	0.74 ± 0.02
ID0	0.72 ± 0.08	1.80 ± 0.35	0.79 ± 0.02
IDD0	0.73 ± 0.08	1.84 ± 0.37	0.80 ± 0.02
IDAOD0	0.73 ± 0.08	1.79 ± 0.38	**0.81** ± **0.02**
U-HPNet	**0.74** ± **0.08**	**1.74** ± **0.34**	0.78 ± 0.02

**Table 5:** Performance comparison with alternative networks for tumour growth using the estimated mean.

	Prediction	Size ↓	Segmentation
	(Bacc)	(MAE)	(Dice)
U-Net	0.64 ± 0.09	2.94 ± 0.43	0.77 ± 0.02
BAYES_TD	0.67 ± 0.08	2.29 ± 0.45	**0.78** ± **0.02**
SPU	0.73 ± 0.08	2.14 ± 0.46	0.77 ± 0.01
P2P_GAN	0.69 ± 0.07	2.62 ± 0.43	0.71 ± 0.02
U-HPNet	**0.74** ± **0.08**	**1.74** ± **0.34**	**0.78** ± **0.02**

**Table 6:** Performance comparison with alternative networks for tumour growth using the estimated distribution.

	Bacc_2std	GED ↓
BAYES_TD	0.46 ± 0.08	0.27 ± 0.03
SPU	0.28 ± 0.06	**0.23** ± **0.02**
GAN-P2P	0.26 ± 0.07	0.25 ± 0.04
U-HPNet	**0.57** ± **0.08**	0.29 ± 0.04

## Data Availability

The data presented in this study are available on request from the corresponding author.

## Supplementary Materials

The following supporting information can be downloaded at: https://www.mdpi.com/article/10.3390/diagnostics12112639/s1. Reference [43] is cited in the supplementary materials.
