# Supplementary material for: Prediction of Lung Nodule Progression with an Uncertainty-Aware Hierarchical Probabilistic Network
Source: Diagnostics (Basel). 2022 Oct 31;12(11):2639. doi: 10.3390/diagnostics12112639 (PMC9689366; doi:10.3390/diagnostics12112639)
Supplement: Supplementary file 1 [file diagnostics-12-02639-s001.zip › diagnostics-1947630-supplementary.pdf]

Supplementary material

S1. Hierarchical probabilistic U-Net framework

This framework [1, 2] relies on a CVAE-like network able to generate consistent segmentations of an image. To do this, the method aims to approximate the posterior distribution of image segmentations via latent features injection and variational inference. In particular, it defines two inter-related subnetworks: the prior and the posterior. Both receive as input the image X to segment, and the posterior receives also the segmentation of the image Y.

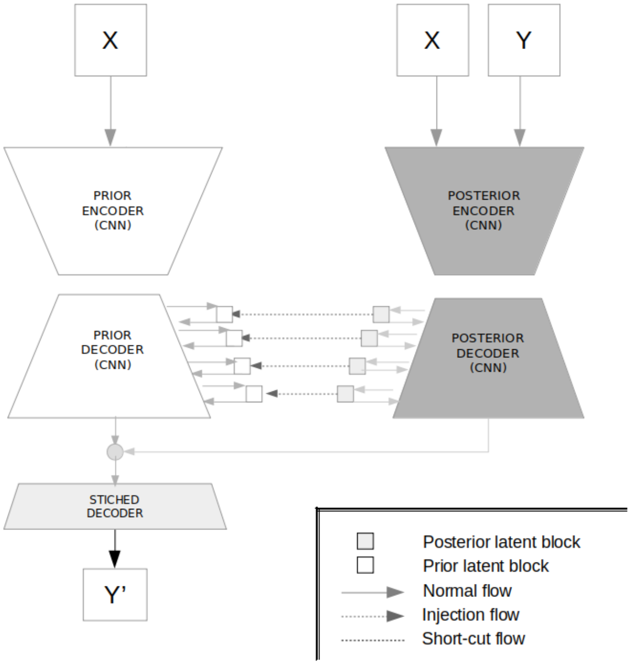

Figure S1: General overview of the HPU network architecture. On the left of the picture we can observe the prior network and on the right the posterior. Both networks have different probabilistic latent blocks interleaved along the decoder component.

The inference process consists on forward-pass the input image, X, through the prior network to obtain a segmentation, Y'. Along the decoder of the prior network, feature activation maps are concatenated with vectors, $\mathrm{z_i}$ ($\mathrm{i \leq L}$, being L the number of latent hierarchies), obtained from sampling different latent distributions interleaved in the decoder. With just another forward-pass of the same input image through the network, a new sample is obtained.

The training aims at minimizing the loss of the reconstructed segmentation, while pulling the variational posterior distribution $Q(.|X,Y)$ of image segmentations, defined by the posterior network, towards the prior distribution $P(.|X)$ of reconstructed segmentations, encoded by the prior network with the latent feature vectors $\mathrm{z_i}$ injected from the posterior network. This is the same as maximizing the evidence lower bound (ELBO) in variational inference, which is equivalent to adding the Kullback-Leibler divergence loss (D_{KL}) between the posterior and the prior distributions, with the reconstruction objective obtained through the cross-entropy loss (represented by the pixel-wise categorical distribution P_{c}) between the prediction Y' (obtained from the prior network with the latent features injected from the posterior) and the target Y.

$$\mathcal{L}_{\mathrm{ELBO}} = \mathbb{E}_{\mathrm{z\sim Q}}[-logP_{\mathrm{c}}(Y|Y')]+$$
$$\beta \sum_{i=0}^{L} \mathbb{E}_{\mathrm{z_{<i}\sim Q}}[D_{\mathrm{KL}}(q(z_{\mathrm{i}}|z_{<\mathrm{i}},X,Y)||p(z_{\mathrm{i}}|z_{<\mathrm{i}},X))]$$

where $\mathbb{E}_{\mathrm{z\sim Q}}$ is the expectation operator, and z a vector sampled from the posterior distribution Q. For this, it is assumed that both P and Q can be decomposed into:

$$P(z_0,...,z_L|X) = p(z_L|z_{<L},X)\cdot ... \cdot p(z_0|X)$$
$$\mathrm{Q}(\mathrm{z}_0,...,z_L|X,Y) = q(z_L|z_{<L},X,Y)\cdot ... \cdot q(z_0|X,Y)$$

To enable a stable training, in [2] β was set to balance both terms of the loss together with a deep supervision to each resolution level of the network. In [1] the authors used the GECO mechanism [3] to dynamically balance both terms of the ELBO, together with online hard-negative mining.

S2. Adaptation of related deep networks to predict lung tumour growth

We built and adapted 4 different state-of-the-art deep architectures to compare the performance of our method, one deterministic (U-Net) and three generative networks (probabilistic U-Net (SPU), the Bayesian dropout (BAYES_TD), and the Pix2Pix (P2P_GAN).

As for the deterministic (or baseline) approach, we used a single U-ResNet like network. This network was trained using a conventional loss function, formed by a pixel-wise binary cross entropy, without any additional configuration.

The first generative method was a Bayesian dropout network (BAYES_TD) following the Bayesian SegNet proposed in [4]. This approach provides a probabilistic pixel-wise semantic segmentation by enabling dropout at inference time. Therefore, this approach aims to find the posterior distribution over the convolutional weights, W, given the observed image I_0 and labels Y, i.e. $p(W|I_0, Y)$. According to the authors, the best configuration was obtained using dropout in the central part of the network. Thus, we followed the same suggestion and we setup dropout (p=0.5) layers in the 3 last encoder and 3 initial decoder blocks of the U-ResNet. This network was trained using pixel-wise binary cross entropy.

The second proposed generative network was the former version of the HPU, the standard probabilistic U-Net (SPU) [5]. This approach goes beyond the notion of reporting a per-pixel probability map, by capturing the co-variances between pixels and providing consistent, structured outputs. To do this, two networks: the prior (having as input a nodule I_0) and the posterior (which also receives the nodule I_1), learn to map the input into a low dimensional latent space that encodes the distribution of all possible segmentation variants for the given input. In particular, we configured a latent vector of 6 features (or dimensions) as in the original paper. This network was trained to maximize the ELBO composed by the pixel-wise binary cross entropy between the predicted and the ground truth segmentation, and the KL-divergence between the posterior and the prior distributions. By sampling on the latent features of the prior network,

this method allows generating multiple segmentations at inference time.

The last generative approach consisted on a conditional GAN named Pix2Pix [6]. The framework allows learning, in a model-free fashion, a mapping between two images. In our case, the two images were a nodule image I_0 and a segmentation image Y at T_1. The proposed network (P2P_GAN) is composed by two networks; a generator formed by U-ResNet configured with dropout (p=0.5) along the decoder (no specific locations were indicated by the authors), and a discriminator composed by the encoder part of a U-ResNet. These two networks learn to generate images that are as similar as real ones, as well as to discriminate between real and fake images that are increasingly similar. This network was trained as suggested by the authors, using the $\mathcal{L}_{\mathrm{cGAN}}$ loss:

$$\mathcal{L}_{\mathrm{cGAN}}(G, D) = \mathbb{E}_{\mathrm{I}_1,\mathrm{Y}}[log D(I_1, Y)] + \mathbb{E}_{\mathrm{I}_0,\mathrm{I}_1,\mathrm{z}}[log(1 - D(I_1, G(I_0, z)))],$$

which represents the sum between the discriminator D loss (i.e. binary cross entropy) of a nodule I_1 and the segmentation ground truth Y, and one minus the discriminator loss of a nodule I_1 and the segmentation Y' produced by the generator at T_1, i.e. $Y' = G(I_0, z)$. Additionally, a second term was added into this loss to figure out the fidelity of the generated samples with the ground truth. Thus, the L1 distance was computed between the generated sample Y' and the ground truth Y. The final loss G^* is as follows:

$$G^* = arg \min_G \max_D \mathcal{L}_{\mathrm{cGAN}}(G, D) + \lambda \mathcal{L}_{\mathrm{L1}}(G).$$

This approach allowed generating multiple samples by adding noise in the form of dropout, applied during training and test time.

S3. Further qualitative reults

Below, we provide further qualitative results of the U-HPNet for the cases B01 (Figure-S2), C94 (Figure-S3) and C99 (Figure-S4) compared with the annotations provided by three radiologists. Additionally, we provide two other cases (Figure-S5) where the U-HPNet fails to predict growth.

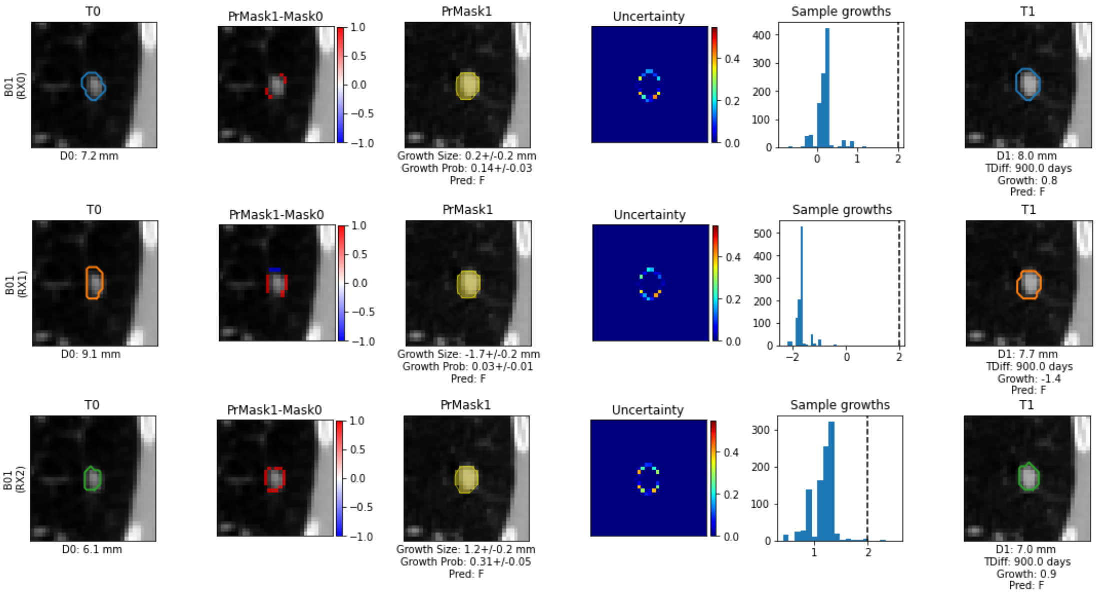

Figure S2: Comparison of ground truth annotations and predictions from the U-HPNet for the tumour case B01. In this figure, the first column is the nodule at T_0 overlapped with the segmentation of a radiologist. The second shows, overlapped with the nodule at T_0, the difference between the ground truth segmentation at T_0 and the estimated mean segmentation at T_1. The third provides, overlapped with the nodule image at T_1, the estimated tumour mean segmentation. The fourth is the estimated uncertainty probability map with the per pixel-standard deviation. The fifth shows the histogram of the (K=1000) estimated tumour diameter growths (dashed line is the 2 mm tumour growth threshold). The last column shows the nodule at T_1 overlapped with the segmentation of the radiologist at T_1

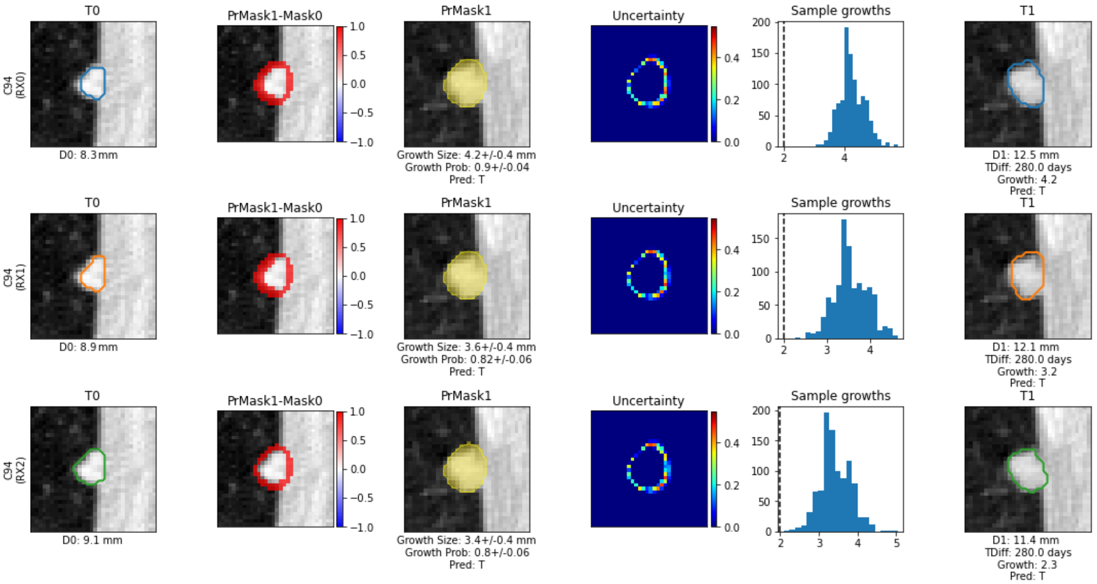

Figure S3: Comparison of ground truth annotations and predictions from the U-HPNet for the tumour case C94.

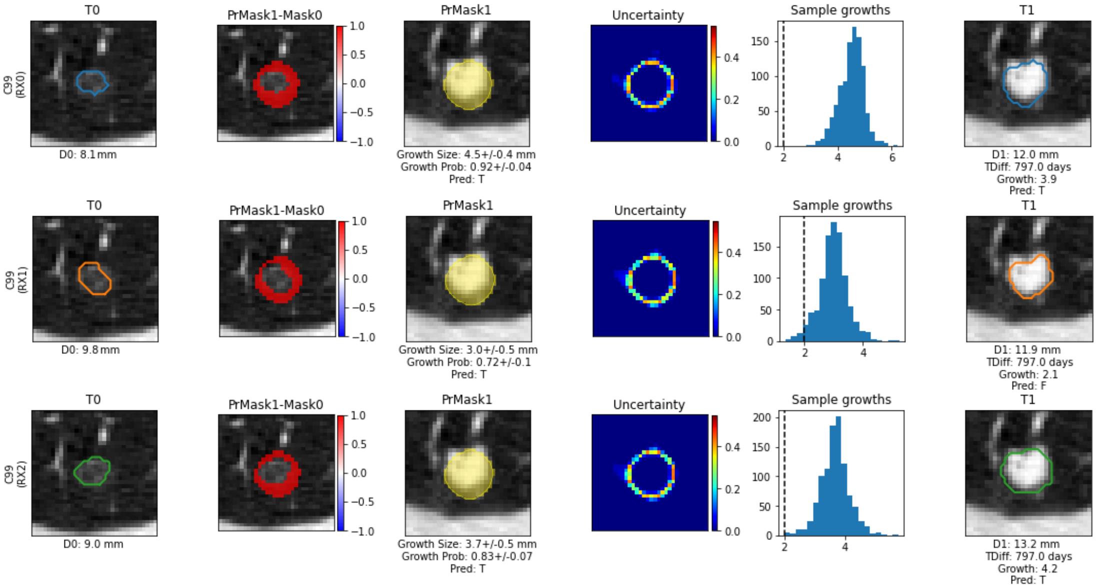

Figure S4: Comparison of ground truth annotations and predictions from the U-HPNet for the tumour case C99.

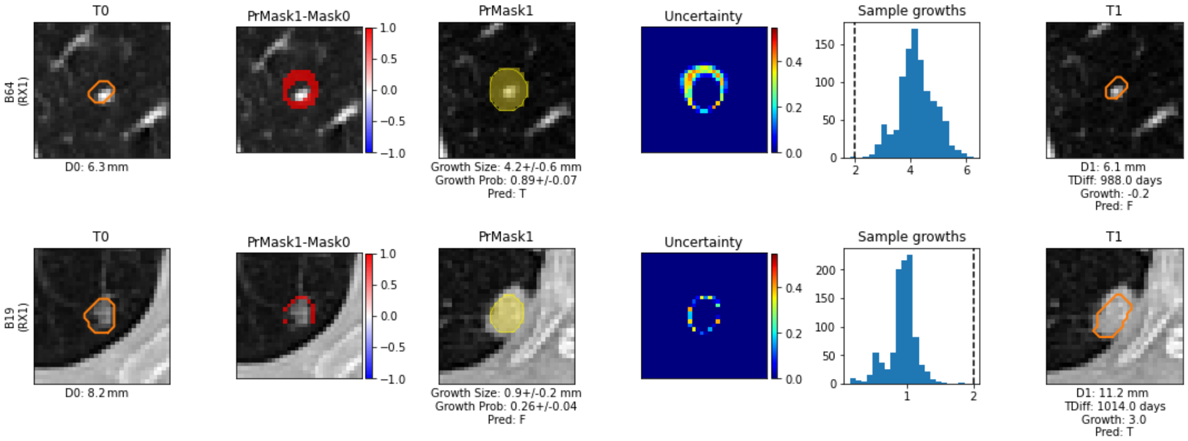

Figure S5: Comparison of ground truth annotations and predictions from the U-HPNet for the tumours B64 and B19. In this example, the network incorrectly predicts growth for these cases.

References

[1] Kohl, S.A.; Romera-Paredes, B.; Maier-Hein, K.H.; Rezende, D.J.; Eslami, S.; Kohli, P.; Zisserman, A.; Ronneberger, O. A hierarchical probabilistic U-Net for modeling multi-scale ambiguities. *arXiv preprint arXiv:1905.13077* **2019**.

[2] Baumgartner, C.F.; Tezcan, K.C.; Chaitanya, K.; Hötker, A.M.; Muehlematter, U.J.; Schawkat, K.; Becker, A.S.; Donati, O.; Konukoglu, E. Phiseg: Capturing uncertainty in medical image segmentation. In Proceedings of the International Conference on Medical Image Computing and Computer-Assisted Intervention. Springer, 2019, pp. 119–127.

[3] Rezende, D.J.; Viola, F. Taming VAES. *arXiv preprint arXiv:1810.00597* **2018**.

[4] Kendall, A.; Badrinarayanan, V.; Cipolla, R. Bayesian segnet: Model uncertainty in deep convolutional encoder-decoder architectures for scene understanding. *arXiv preprint arXiv:1511.02680* **2015**.

[5] Kohl, S.; Romera-Paredes, B.; Meyer, C.; De Fauw, J.; Ledsam, J.R.; Maier-Hein, K.; Eslami, S.A.; Rezende, D.J.; Ronneberger, O. A probabilistic U-Net for segmentation of ambiguous images. In Proceedings of the Advances in Neural Information Processing Systems, 2018, pp. 6965–6975.

[6] Isola, P.; Zhu, J.Y.; Zhou, T.; Efros, A.A. Image-to-image translation with conditional adversarial networks. In Proceedings of the Proceedings of the IEEE Conference on Computer Vision and Pattern Recognition, 2017, pp. 1125–1134.
